# A Novel Zebrafish Xenotransplantation Model for Study of Glioma Stem Cell Invasion

**DOI:** 10.1371/journal.pone.0061801

**Published:** 2013-04-16

**Authors:** Xiao-jun Yang, Wei Cui, Ai Gu, Chuan Xu, Shi-cang Yu, Ting-ting Li, You-hong Cui, Xia Zhang, Xiu-wu Bian

**Affiliations:** 1 Institute of Pathology and Southwest Cancer Center, Southwest Hospital, Third Military Medical University, Chongqing, China; 2 Key Laboratory of Tumor Immunopathology of Ministry of Education of China, Third Military Medical University, Chongqing, China; University of Bari Medical School, Italy

## Abstract

Invasion and metastasis of solid tumors are the major causes of death in cancer patients. Cancer stem cells (CSCs) constitute a small fraction of tumor cell population, but play a critical role in tumor invasion and metastasis. The xenograft of tumor cells in immunodeficient mice is one of commonly used *in vivo* models to study the invasion and metastasis of cancer cells. However, this model is time-consuming and labor intensive. Zebrafish (*Danio rerio*) and their transparent embryos are emerging as a promising xenograft tumor model system for studies of tumor invasion. In this study, we established a tumor invasion model by using zebrafish embryo xenografted with human glioblastoma cell line U87 and its derived cancer stem cells (CSCs). We found that CSCs-enriched from U87 cells spreaded *via* the vessels within zebrafish embryos and such cells displayed an extremely high level of invasiveness which was associated with the up-regulated MMP-9 by CSCs. The invasion of glioma CSCs (GSCs) in zebrafish embryos was markedly inhibited by an MMP-9 inhibitor. Thus, our zebrafish embryo model is considered a cost-effective approach tostudies of the mechanisms underlying the invasion of CSCs and suitable for high-throughput screening of novel anti-tumor invasion/metastasis agents.

## Introduction

Recurrence and metastasis of solid tumors are the most common causes of cancer-related deaths [1]. Tumor metastasis is a complex, dynamic, and multi-step process, including tumor cell intravasation into the circulation, scattering to distant organs, extravasation into the parenchyma for colonization, and outgrowth of secondary lesions [2], [3]. Invasiveness is the basic characteristics of metastatic tumor cells.

Cancer stem cells (CSCs), or tumor initiating cells, constitute a minor population of cancer cells in tumor mass. CSCs are responsible for tumorigenicity, and play an important role in tumor metastasis [4]–[12]. CSCs have been isolated and characterized from more than 20 cancer types [9], [13], [14]. Although studies have been focused on the role of CSCs on tumor invasion and metastasis, the mechanisms underlying the stemness of such cells remain poorly understood.

One of the widely-used *in vivo* models to invstigate the invasion or metastasis of cancer cells or CSCs is xenograft in immunodeficient mice. However, this model is often considered time-consuming and labor intensive. Zebrafish (*Danio rerio*) and their transparent embryos recently emerge as another promising xenograft tumor model system in tumor therapeutic drug screening [15], [16]. Zebrafish provides unique features for investigating tumor development, angiogenesis, invasion and metastasis. The model has shown advantages in its simplicity for genetic manipulation, inexpensive to maintain, easy visualization of internal structures, and rapid embryonic development. As a vertebrate animal, zebrafish model offers higher levels of physiologic and genetic similarities to mammals [17], [18]. Several reports have shown that human tumor cells proliferate and interact with the vessel tissues in zebrafish embryos [19]–[22]. Malignant glioma is the most common brain cancer, with a highly invasive behavior. Zebrafish embryos offer an excellent model to investigate the mechanism of glioma cell and CSC invasion and spread *in vivo*.

In this study, we established a novel zebrafish embryo xenograft model to analyze the invasion and spread of human glioma CSCs (GSCs). We demonstrate that GSCs derived from human glioblastoma cell line U87 possessed a highly invasive phenotype. We also reveal that the highly invasive capability of U87 GSCs was associated with enhanced expression MMP-9. Moreover, an MMP-9 inhibitor suppressed the invasion/spread of GSCs in zebrafish embryos. Our study indicates that zebrafish embryo model is a cost-effective system for better understanding the mechanisms utilized by CSCs for invasion and spread, as well as for high-throughput screening of novel anti-cancer agents.

## Materials and Methods

### Ethics Statement

This study was carried out in strict accordance with the recommendations in the Guide for the Care and Use of Laboratory Animals of the Third Military Medical University (TMMU). The protocol was approved by the Committee on the Ethics of Animal Experiments of Southwest Hospital, TMMU (No. 201110-1).

### Animal care and handling

Zebrafish (Danio rerio) and transgenic zebrafish Tg (fli1:EGFP)y1 (a gift from Dr. Lu Wen, Sun Yat-Sen Univeristy, Guangzhou, China) were raised as previously described [23]. The fish were kept at 28°C in aquaria (ESEN Environ Science, China) with day/night light cycles. The embryos were raised at 28°C in E3 egg water until the desired developmental stages [24]. Embryos raised beyond 24 hrs post-fertilization (hpf) were treated with phenylthiourea (PTU; 0.003%, w/v; Sigma, USA).

### Cell culture and establishment of a stable red fluorescent protein (RFP) expressing glioma cell line

U87 cells were obtained from the American Type Culture Collection (ATCC) and maintained in Dulbecco's modified Eagle's medium (DMEM) (Gibico, USA), containing 10% fetal bevine serum (FBS) (Hyclone, USA) and 1∶100 Pen/Strep (Invitrogen, USA). To establish a stable RFP-expressing U87 cell line, the cells were transfected with a pcDNA3.1(+)-RFP vector by Lipofectamine 2000™ (Invitrogen, USA). G-418 selection (400 µg/ml, Invitrogen, USA) was performed 48 hrs later. The stable RFP expressing U87 cells were grown in DMEM cell culture medium containing 10% FBS, Pen/Strep antibiotics and G-418 (200 µg/ml).

### The Isolation of U87 glioma sphere cells

The isolation of U87 sphere cells was performed as described previously [25]. Briefly, U87 cells were seeded in a 24-well plate at 2×10^4^ cells/well for 12–18 hrs. Thereafter, 250 µl culture medium were replaced with an equal volume of serum-free neural stem cell medium containing DMEM/F12 (Gibco, USA), B27 (Gibco, USA), recombinant human epidermal growth factor (rhEGF, 20 ng/ml; Sigma, USA), basic fibroblast growth factor (bFGF, 20 ng/ml; Upstate, USA), leukemia inhibitory factor (LIF, 10 ng/ml; Chemicon, USA), insulin (4 U/L; Sigma, USA), with or without vincristine (5 ng/ml; Hualian Pharmaceutical Co. China). This procedure was repeated every 24 hrs until several primary tumor spheres were visible under microscopy. At this point, the culture medium was removed and refilled with 1 ml fresh serum-free neural stem cell medium.

The primary tumor spheres were dissociated and single cells were seeded in 24-well plates in 1 ml/well of serum-free neural stem cell medium. The culture medium was changed every 3 days. Cells from secondary spheres derived from single cells of primary spheres were used for xenograft injection. Stem cell markers such as CD133, Nanog and Sox-2 were examined using quantitative real-time PCR (qRT-PCR) and immunofluorescence staining. Adherent U87 cells were used as controls for the xenograft injection.

### Quantitative real-time PCR (qRT-PCR)

Total RNA was extracted from tumor cells using Trizol™ Reagent (Invitrogen, USA) according to the manufacturer's protocol. qRT-PCR was performed by using SYBR PrimeScript RT-PCR kit (TaKaRa, Japan) on a Rotor-Gene 6000 real-time genetic analyzer (Corbett Life Science, USA) according to manufacturer's instructions. The primer sequences of MMP-2, MMP-9, and GAPDH of internal control are: MMP-2 forward primer: 5′agaagttctttggactgcccc3′, reverse primer: 5′caggtgtgtagccaatgatcc3′; MMP-9 forward primer: 5′tgtaccgctatggttacactcg3′, reverse primer: 5′ggcagggacagttgcttct3′; and GAPDH forward primer: 5′tgcaccaccaactgcttagc3′, reverse primer: 5′ggcatggactgtggtcatgag3′. The PCR protocol included denaturation program (95°C for 2 min), followed by 40 cycles of amplification and quantification program (95°C for 5 sec, 55°C–57°C for 30 sec) and melting curve program (55°C–95°C, with 0.5°C increment each cycle). Each sample was replicated three times.

### Microinjection of glioma cells

The RFP-expressing U87 cells were washed, re-suspended in PBS, were then sorted by a fluorenscence flow cytometry (FACS Aria™ II; BD Biosciences, USA). Fluorescence-emitting positive cells were collected in a sterile tube.

For microinjection of tumor cells into the embryos, Tg (*fli1*:EGFP)^y1^ zebrafish embryos of 2 days post-fertilization (dpf) were dechorionated and anesthetized with tricaine (MS-222; Sigma, USA). The desired numbers of U87-RFP cells were injected into the middle of embryonic yolk *sac* region using a Pneumatic Pico-Pump Injector (PLI-100; Harvard Apparatus, USA) with an injection needle (World Precision Instruments Inc., USA) pulled by a P-97 Flam/Brown Micropipette Puller (Sutter Instruments Co., USA). After injection, embryos with fluorescent cells outside the desired injection region were excluded from further analysis. The injected cell number was measured by fluorescence intensity with an ImageJ software (NIH, Bethesda, USA). The embryos injected with same volume of medium in the absence of tumor cells were defined as control embryos. The embryos were incubated at 35°C.

### Whole mount immunofluorescence of zebrafish embryos

After microinjection, embryos were examined under an Olympus SZX-10 fluorescent microscope at 2 days post-injection (dpi). All embryos were handled identically and their exposure to incidental light was minimized in 3% methylcellulose (Sigma, USA). Both bright field and fluorescent images were captured with a QImaging digital camera controlled with Image-Pro Express software and processed by Adobe Photoshop CS2 (Adobe, USA).

### Immunofluorescence staining and confocal microscopy

Confocal microscopy was used to determine the invasive characteristic of tumor cells in Tg (*fli1*:EGFP)^y1^ embryos. The injected embryos at desired stages were fixed in 4% paraformaldehyde (PFA) in PBS at room temperature for 2 hrs. The fixed embryos were then infiltrated with 40% sucrose in PBS at room temperature for 2 hrs, embedded in Tissue-Tek O.C.T. compound (Sakura, Japan), and cryosectioned at 35 µm thickness. The cryosections were washed with PBS and incubated with 2% BSA in PBS (blocking buffer). The sections were then incubated with polyclonal anti-CD133 antibody (1∶300, Abcam, England) in blocking buffer at 4°C overnight. After six washes with PBST buffer (1×PBS containing 0.5% Triton X-100) at room temperature for 15 min each, sections were incubated with Cy5-conjuncted secondary antibody (Jackson ImmunoResearch, USA). The samples were then washed four times, mounted in mounting medium (Vector Lab, USA), and sealed under coverslips with nailpolish. The prepared samples were observed and photographed under a Leica TCS-SP5 laser confocal scanning microscope. For CD133 positive/negative cells detection, we analyzed all of the cells with CD133 immunofluorescence staining within random vision fields. Three-dimensional reconstruction was analyzed by Leica LAS AF software.

### Flow cytometry

The percentage of CD133-expressing cells in U87 sphere cells was analyzed by flow cytometry. 1×10^7^ U87 sphere cells were re-suspended with 80 µl PBS and 20 µl Fc Blocking Reagent (Miltenyi Biotec., Germany), and then stained with 10 µl anti-CD133/2 (293C3)-APC antibody or isotype control monoclonal IgG2b-APC antibody (Miltenyi Biotec., Germany) for 30 min at 4°C in the dark. Analysis of fluorescence intensity was performed by flow cytometry on FACS Calibur (BD, USA). For isolating CD133-positive cells, U87 sphere cell suspension was pelleted and stained with anti-CD133/2 (293C3)-APC antibody for 30 min at 4°C. Then CD133-positive cells were sorted by flow cytometry on the FACS Aria II (BD, USA). Staining with 7AAD (BD, USA) was used for flow cytometric exclusion of dead cells.

### Western blot analysis

To analyze the protein levels, the cells were washed by PBS for three times and then lysed (1% Triton X-100 in PBS buffer with protease inhibitor, Roche, USA) for 30 min on ice. After incubation, the cell lysates were microcentrifuged at 13,000 rpm for 10 min. The supernatant was mixed with 6× SDS sample buffer and boiled for 10 min, and electrophoresed at 80 V through a 10% polyacrylamide-SDS denaturing gel. Separated proteins were electrotransferred to nitrocellulose, and the expression of CD133, MMP-2 or MMP-9 protein was detected with the desired antibodies including monoclonal anti-CD133 antibody (1∶1000, Abcam, England), polyclonal anti-MMP-2 antibody (1∶1000, Abcam, England), polyclonal anti-MMP-9 antibody (1∶1000, Abcam, England), and monoclonal anti-β-actin antibody (1∶2000, Genscript, USA), respectively.

### Statistical analysis

All results were analyzed by SPSS10.0 statistical software and presented as the arithmetic mean ± SE. Student's *t* test was performed for statistical analysis.

## Results

### Establishment of glioma xenograft in zebrafish embryos to study GSC invasion

Based on our reported angiogenesis model [26], we extended study to examine GSC invasion and spread in zebrafish embryos. Glioblastoma cell line U87 was stably transfected with pCDNA3.1(+)-RFP plasmid to produce fluorescence with low background [27]. Also, Tg (*fli1*:EGFP)^y1^ fish stably expressing EGFP in all blood vessels throughout embryogenesis clearly, were utilized to demonstrate interactions between metastatic tumor cells and host vessels.

**Figure 1:**
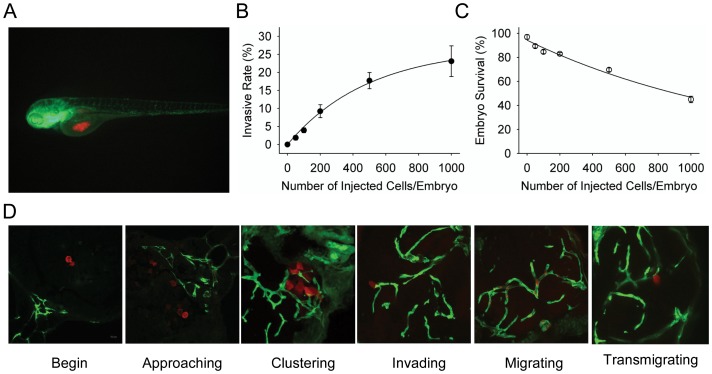
The establishment of U87 glioma sphere cell invasion model in zebrafish embryos. A. Dual color confocal image shows that U87 sphere cells (RFP labeled, red) were microinjected into the middle of yolk *sac* within Tg (*fli1*:EGFP)^y1^ transgenic zebrafish embryos (EGFP labeled, green). B. Different numbers of U87-RFP glioma sphere cells were microinjected into Tg (*fli1*:EGFP)^y1^ embryos (n = 300 in each group), and the percentage of embryos with invasive tumor cells was quantitated. C. The survival rate of Tg (*fli1*:EGFP)^y1^ zebrafish embryos microinjected with different numbers of U87-RFP glioma sphere cells (n = 300 in each group). D. Representative dual color confocal images of RFP-labeled U87 sphere cells within Tg (*fli1*:EGFP)^y1^ zebrafish embryos at the different invasive stages. Red: RFP-labeled U87 sphere cells; Green: Tg (*fli1*:EGFP)^y1^ microvessels.

To characterize GSCs, we isolated sphere cells from U87 glioblastoma cell line [28] and performed microinjection of GSCs into the middle of embryonic yolk *sac* (Figure 1A) [27]. U87 sphere GSCs displayed invasive and metastatic behavior within zebrafish embryos. Quantitative analysis indicates that injection at 2 dpi with increasing number of U87 sphere cells resulted in increasing embryos with an invasive phenotype. Also, injecting higher cell numbers increased the mortality of embryos (Figure 1B, 1C and Table S1). When 500 U87 sphere cells were injected into each embryo, the survival rate of the embryos was 68%. Thus, injection of 300 tumor cells into 2 dpf embryos was adopted for measurement of both survival and invasion rates.

In live embryos, 14.1% (29/206), 20.2% (39/193) and 19.2% (34/177) injected with U87 sphere cells showed invasive phenotype at 1, 2, or 3 dpi. At 3 dpi, higher mortality rate resulted in fewer remaining live embryos with invasive tumor cells. Thus, 2 dpi was chosen for subsequent experiments.

### Glioma sphere cells invade *via* vessels within zebrafish embryos

U87 sphere cells were injected into the yolk *sac* of Tg (*fli1*:EGFP)^y1^ embryos at 2 dpf. The embryos injected with cancer cells were then fixed, cryosectioned and observed under a confocal microscope to track the metastases and dynamic changes in tumor cell-vascular interface. As shown in Figure 1D, many tumor cells moved to embryonic vasculature after microinjection in the yolk *sac*. Subsequently, RFP-labeled cells clustered around EGFP-labeled vasculature of the embryos. Some of the tumor cells transformed to a protrusive appearance to interact with the host vessel (Figure 1D). Eventually, the tumor cells invaded the vessels to establish distant metastases *via* embryo vessels.

### The invasiveness of glioma cells is correlated with CD133 expression

**Figure 2:**
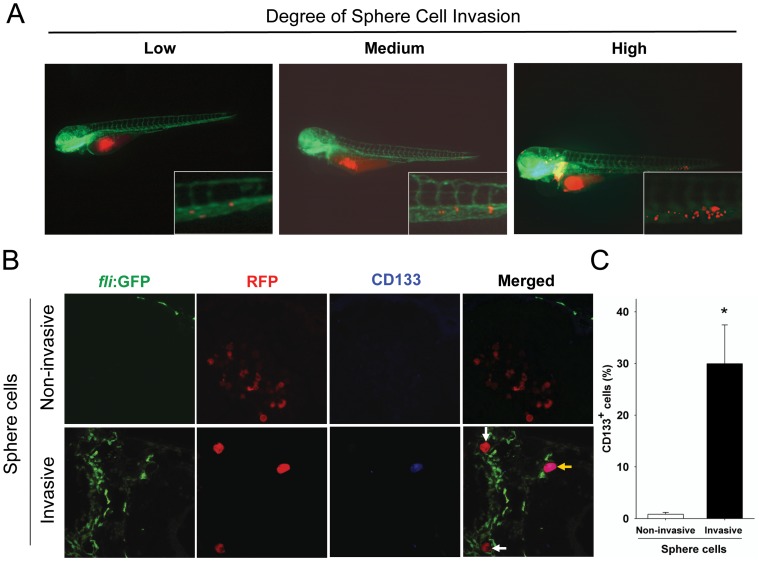
Invasive U87 sphere cells express CD133. A. U87 sphere cells with various invasion capability within zebrafish embryos. The extent of invasion was classified in three degrees: Low: less than 5 migrated cells; Medium: 5–20 migrated cells; High: more than 20 migrated cells. Representative images at higher magnification show the invasive RFP-labeled U87 sphere cell masses (red) in the tail region of the embryos *via* EGFP-labeled host vessels (green). B. Detection of CD133 expression on non-invasive and invasive U87 sphere cells at 2 dpi by immunofluorecent staining. All of U87 sphere cells within injected embryos were stained with monoclonal anti-CD133 antibody (1∶300) and examined by confocal microscopy. Green: Tg (*fli1*:EGFP)^y1^ microvessels; red: RFP-labeled U87 sphere cells; blue: CD133 positive U87 cells. C. Quantitative analysis of CD133-expressing cells in non-invasive cell group (n = 713) and high-invasive cell group (n = 175) at 2 dpi. (*p*<0.001).

We next classified the invasiveness of U87 sphere cells into low (less than 5 migrating tumor cells per embryo), medium (between 5 and 20 migrating tumor cells per embryo), or high (more than 20migrating tumor cells per embryo) as previously described [27] (Figure 2A).

Because tumor cells were injected into the middle of the embryo yolk *sac* where there were no EGFP-labeled vessels in the naïve state, we regarded tumor cells without physical interaction with EGFP-labeled host vessels as non-invasive (*upper panel*, Figure 2B). Tumor cells attached to EGFP-labeled vasculature were defined as invasive (*bottom panel*, Figure 2B).

U87 sphere cells were enriched with GSCs [25], which might exhibit higher invasive capability than differentiated U87 cells. To verify the issue, CD133, a well-defined GSC marker, was examined. CD133^+^ cells were almost undetectable in the non-invasive cell subpopulation (RFP labeled cells in Figure 2B, *upper panel*). In contrast, both CD133^+^ cells (yellow arrow in Figure 2B, *lower panel*) and CD133^−^ U87 sphere cells (white arrows in Figure 2B, *lower panel*) were found in the invasive cell subpopulation. Calculation of the percentage of CD133^+^ cells in invading cells revealed that approximately 30% invasive glioma cells expressed CD133 (53/175), while less than 1% non-invasive glioma cells were CD133 positive (6/713) (Figure 2C). These results suggest that CD133-expressing glioma cells are the dominant invasive population in zebrafish embryos.

### GSCs display extremely high invasiveness in zebrafish embryos

**Figure 3:**
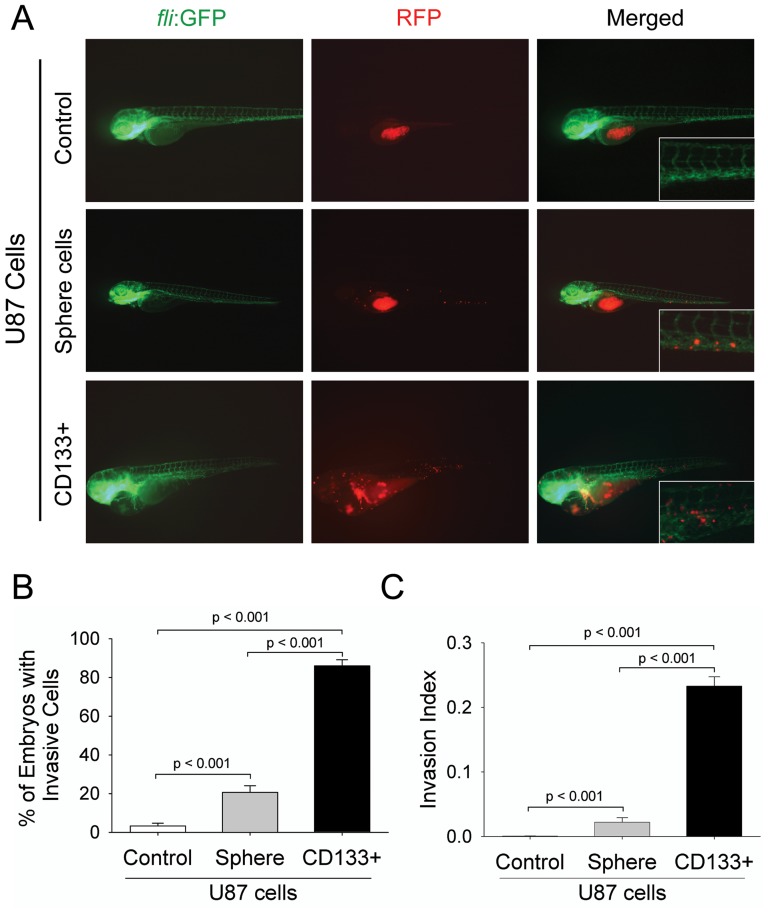
CD133+ U87 GSCs are highly invasive within zebrafish embryos. A. Representative images of the invasion of differentiated U87 cells, U87 sphere cells, and CD133+ U87 GSCs within the injected embryos at 2 dpi. The images at higher magnification show the invasive RFP-labeled cell masses at tail region of embryos *via* host vessels. B. The percentage of the embryos with invasive cells injected with RFP-labeled differentiated U87 cells, U87 sphere cells, and CD133^+^ U87 GSCs. The data were obtained from three replicate experiments of 50 injected embryos in each experiment: n = 124 for live embryos injected with differentiated U87 cells, n = 121 for embryos injected with U87 sphere cells, and n = 120 for embryos injected with CD133^+^ cells C. The percentage of invasive cells within total injected cells (Invasion Index) in the embryos. All injected cells including invasive or non-invasive cells within zebrafish embryos were evaluated by ImageJ software through fluorescence intensity. n = 37200 (300 injected cells per embryo among 124 live embryos) for differentiated U87 cell group, n = 36300 (300 injected cells per embryo among 121 live embryos) for U87 sphere cell group, and n = 36000 (300 cells per embryo among 120 live embryos) for CD133^+^ U87 GSCs group (*p*<0.001).

It has been shown that CD133 positive U87 cells were highly invasive *in vitro* [29]. We sorted CD133^+^ cells from U87 sphere cells to test their invasiveness in Tg (*fli1*:EGFP)^y1^ embryos. As shown in Figure 3A, CD133^+^ U87 cells microinjected into Tg (*fli1*:EGFP)^y1^ embryos at 2 dpi exhibited much higher level of invasion as compared with differentiated U87 cells (*top panel*) or unsorted sphere cells (*middle panel*). Unlike differentiated U87 or unsorted sphere cells, more CD133^+^ cells migrated to the edge of embryonic yolk *sac*, distant from the original injection region (Figure 3A, *bottom panel*). Notably, embryos injected with CD133^+^ cells displayed partial developmental disruption, including pericardial edema and heart linearization (Figure 3A, *bottom panel*), possibly due to blood vessel blockade by a large number of invasive GSCs in the vasculature. Quantitative analyses indicated that embryos injected with CD133^+^ cells contained higher number of invasive cells (85.1%) than those injected with differentiated U87 cells (3.2%) or unsorted sphere cells (20.7%) (Figure 3B). Similarly, the invasion index (the percentage of invasive cells in total injected cells among live injected embryos) in embryos injected with CD133^+^ cells was markedly higher than those injected with unsorted U87 sphere cells or differentiated U87 cells (Figure 3C). Moreover, there was a significantly increased percentage of embryos that contained medium and highly invasive tumor cells when they were injected with CD133^+^ U87 cells (Table 1). These results indicate a more highly invasive behavior of GSCs in zebrafish embryos.

**Table 1:** Quantitation of invading tumor cells within zebrafish embryos injected with U87 cells.

Groups (No. of injected cells)	Total no. of injected embryos	No. of injected embryos with invasive cells (mean±SD)			Dead
		Low-invasion	Medium-invasion	High-invasion	
U87 control (n = 300)	150	3.2±1.4%	0	0	26
U87 sphere cells (n = 300)	150	6.6±2.6%	8.3±1.4%	5.8±3.0%	29
U87 CD133-expressing cells (n = 300)	150	15.0±2.3%	31.7±2.2%	39.2±2.5%	30

U87 sphere cells and U87 CD133^+^ GSCs. The data were obtained from three replicate experiments of 50 injected embryos for each experiment.

### CD133 expression on GSCs is reduced after invasion

**Figure 4:**
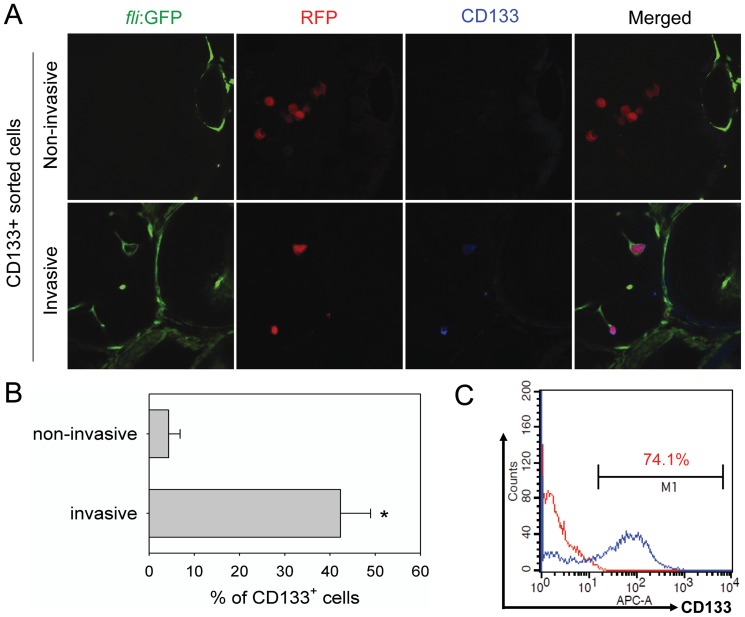
CD133 expression on GSCs is reduced after spread in zebrafish embryos. A. CD133 expression examined on non-invasive or invasive cells in the embryos injected with CD133^+^ U87 GSCs. All CD133^+^ U87 GSCs within injected embryos were stained with a monoclonal anti-CD133 antibody (1∶300) and examined by confocal microscopy. Green: EGFP-labeled endothelial cells; red: RFP-labeled U87 sphere cells; blue: CD133^+^ U87 GSCs. B. The percentage of CD133^+^ cells in non-invasive cells or invasive cells at 2 dpi (n = 300 each group). C. Detection of CD133^+^ cell percentage in sorted CD133^+^ U87 cells in preparation for microinjection by flow cytometry (*p*<0.001).

CD133 expression on non-invasive and invasive tumor cells within embryos after injection was examined at 2 dpi (Figure 4A). Non-invasive U87 cells remaining in the yolk *sac* were CD133 negative (Figure 4A, *upper panel*). However, many invasive cells, localized in the EGFP-labeled embryonic vasculature, were CD133 positive (Figure 4A, lower panel, *yellow arrow*). Quantification shows that only 4.3% (13/300) tumor cells remaining at primary injection site (non-invasive cells) were positive for CD133, whereas 42.3% (127/300) invasive cells spread to distant sites expressed CD133 (Figure 4B). Considering the fact that sorted cells at the time of microinjection were 74% CD133 positive (Figure 4C). The results suggest a reduction of CD133 expression by GSCs during the invasion process.

### MMP-9 mediates the invasion of GSCs

**Figure 5:**
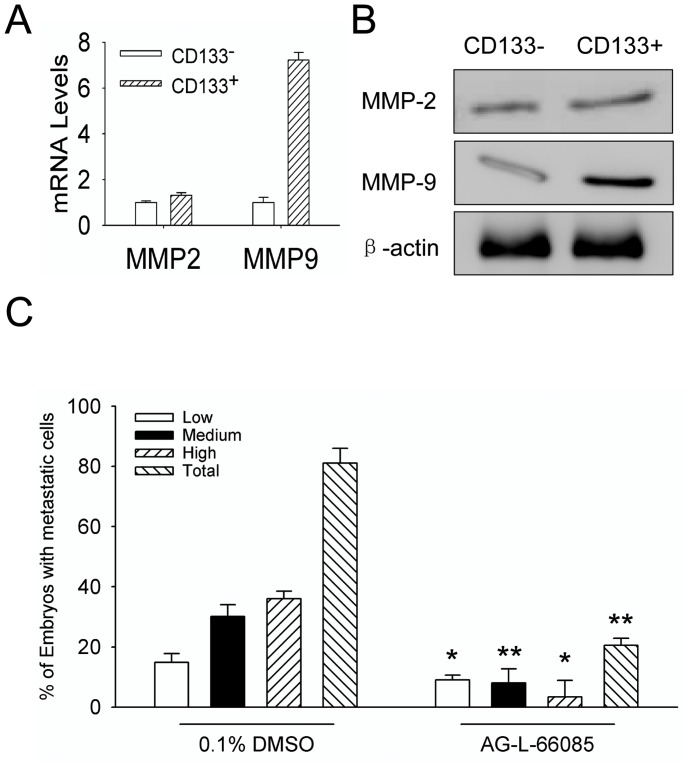
MMP-9 mediates invasion and spread of CD133^+^ U87 GSCs in zebrafish embryos. A. The MMP-2 and MMP-9 RNA in CD133^−^ U87 cells and CD133^+^ U87 GSCs were examined by qRT-PCR. B. The MMP-2 and MMP-9 proteins in CD133^−^ U87 cells and CD133^+^ U87 GSCs examined by Western blot. C. The inhibitory effect of MMP-9 inhibitor (AG-L-66085) on the invasion of CD133^+^ U87 GSCs within zebrafish embryos. The embryos xenografted with CD133^+^ U87 GSCs were treated with 2 µM AG-L-66085 or DMSO control. The percentages of invasive cells in injected embryos (low, medium, or high-invasion) were measured at 2 dpi. The data were obtained from three replicate experiments with the number of embryos: n = 123 for DMSO control group, n = 119 for MMP-9 inhibitor group, and n = 144 for negative control group (*p*<0.001).

MMP-2 and MMP-9 were reported to be associated with the invasion and metastasis of many malignant tumors [29], [30]. Therefore, we examined the function of MMP-2 or MMP-9 in zebrafish embryos injected with U87 cells. The mRNA (Figure 5A) and protein (Figure 5B) of MMP-9 were much higher in CD133 positive GSCs than in CD133 negative cells, with MMP-2 at similar levels (Figure 5). We then treated the embryos with an MMP-9 inhibitor (2 µM, AG-L-66085) (Santa Cruz Biotech) after injection of tumor cells and examined the invasiveness of tumor at 2 dpi. As shown in Figure 5C, the invasion/spread of U87 sphere cells within embryos was significantly inhibited by the MMP-9 inhibitor. The percentage of embryos with invasive cells after treatment with the MMP-9 inhibitor was 20% (24/119) compared with approximate 60% embryos (68/123) without treatment (Figure 5C). No apparent defect was observed in the development of the embryos treated with drug vehicle, or the MMP-9 inhibitor (Data not shown). Our results indicate that MMP-9 plays an important role in the invasion of malignant glioma cells in zebrafish embryos.

## Discussion

Tumor invasion and metastasis constitute a major problem in the treatment of cancer patients. About 30% of patients with newly diagnosed solid tumors already have clinically detectable metastasis. As the most common primary brain tumors, the aggressive invasion by malignant glioma cells into surrounding normal brain tissues has been recognized as an important cause for relapse after surgical excision [31]. Despite the improvement in therapeutic methods in recent years, the median survival time for glioblastoma is still no more than 14 month after diagnosis [32]. During the past decade, emerging evidence supports the notion that CSCs are responsible for tumor development including tumor initiation, invasion, angiogenesis, therapy resistance, and recurrence [33]–[35]. GSCs have been demonstrated to be highly invasive [36], [37]. In fact, invasive glioma cells show tumor stem cell characteristics with neurosphere formation ability and tumorigenicity [34], [38]. However, the molecular mechanisms for invasion and spread of GSCs *in vivo* have not been fully understood.

Zebrafish embryos are suggested as an alternative *in vivo* model for studying the invasion and metastasis of tumor cells [27]. Comparing with mouse models, zebrafish embryo xenograft model has advantages for studying GSC invasion: (1) It is easy to continuously monitor the process of tumor cell invasion/spread in a transparent organ; (2) transgenic zebrafish embryos such as Tg (*fli1*:EGFP)^y1^ offer better examination of interaction between host vessels and tumor cells; (3) the observation period in zebrafish embryos is much shorter than in mice, possible to observe the entire invasion process within two days with sufficient clarity; (4) less tumor cells are required in this model for observations, particularly valuable for studies of GSCs, which are difficult to obtain in high numbers; (5) the model is also suitable for high throughput screening of anti-cancer agents.

CD133 positive cell subpopulation in malignant glioma has been reported to be highly tumorgenic after transplantation in immune-deficient mice [39]. The presence of such cells may explain clinical features of malignant gliomas, such as invasion, relapse and therapy resistance [40]. However, the transformation of GSCs during tumor invasion process *in vivo* has not been well studied. We found that GSCs are essential for gliobastoma cell invasion within zebrafish embryos. In contrast, less than 1% embryos injected with differentiated U87 cells contained invasive tumor cells. These observations are in agreement with our previous observation of invasion and spread of GSCs *in vitro* [25], [38]. CSCs have been notorious for their plasticity [41]. We hypothesized that some CD133^+^ cells injected into zebrafish embryos may differentiate into CD133- cells and lose their potential of invasion. Meanwhile, most CD133^+^ cells may spread to distant regions *via* vasculature but still remain GSC property. Our findings revealed that the transformation and differentiation of GSCs may play an important event in the process of glioma cell invasion/spread *in vivo*. However, the mechanisms regulating such transformation of GSCs require further investigation.

The invasion of malignant glioma require multiple factors. Previous reports suggested that the expression and activation of MMP family proteins may be associated with malignant glioma invasion. More highly malignant glioma express increased levels of MMP-2 and MMP-9. Our results revealed that MMP-9 was highly expressed by CD133^+^ cells and there is a correlation between the GSC stemness and the expression of MMP-9. Therefore, MMP inhibitors, such as integrins and BCNU, have been effective to suppress tumor invasion. In our model, an MMP-9 inhibitor, AG-L-66085, reduced the invasion/spread of CD133^+^ cells within zebrafish embryos.

In summary, we have established a novel GSC xenograft model in zebrafish embryos for evaluation of glioma invasion and spread. This model not only allows for better investigation of the mechanistic basis of the tumorigenic and invasion properties of glioma cells, but also constitutes a platform for studies of other tumors and for screening anti-cancer therapentic agents.

## Supporting Information

Table S1 **The number of zebrafish embryos used in different injection groups.**
(DOC)
