# Supplementary material for: A Novel Zebrafish Xenotransplantation Model for Study of Glioma Stem Cell Invasion
Source: PLoS One. 2013 Apr 16;8(4):e61801. doi: 10.1371/journal.pone.0061801 (PMC3628966; doi:10.1371/journal.pone.0061801)
Supplement: Table S1 — The number of zebrafish embryos used in different injection groups. (DOC) [file pone.0061801.s001.doc]

**Table S1**  The number of zebrafish embryos used in different injection groups

| Groups  (No. of injected cells) | | No. of embryos | | | |
| --- | --- | --- | --- | --- | --- |
|  |  | Total no. of injected  embryos | Live | | Dead |
|  |  |  | Invasive | Non-invasive |  |
| Test for suitable no. of injected U87 cells/embryo | |  | | | |
|  | 0 | 300 | 0 0 | | 13 |
|  | 50 | 300 | 5 | 263 | 32 |
|  | 100 | 300 | 10 | 244 | 46 |
|  | 200 | 300 | 23 | 226 | 51 |
|  | 500 | 300 | 37 | 172 | 91 |
|  | 1000 | 300 | 31 | 104 | 165 |
| Test for invasion ability of tumor cells/embryo | |  | | | |
|  | Sorted CD133+ U87 cells (n=300) | 150 | 103 | 17 | 30 |
|  | U87 sphere cells (n=300) | 150 | 25 | 96 | 29 |
|  | Control U87 cells (n=300) | 150 | 4 | 120 | 26 |
|  | Control (n=0) | 150 | 0 0 | | 11 |
| Test for inhibition of MMP-9 | |  | | | |
|  | 0.1% DMSO (n=300) | 150 | 68 | 55 | 27 |
|  | AG-L-66085 (n=300) | 150 | 24 | 95 | 31 |
|  | Control (n=0) | 150 | 0 0 | | 6 |
